# Cultural Challenges: The Most Important Challenge of COVID-19 Control Policies in Iran

**DOI:** 10.1017/S1049023X20000710

**Published:** 2020-05-21

**Authors:** Hamid Jafari, Majid Amiri Gharaghani

**Affiliations:** 1. Assistant Professor of Health in Disasters and Emergencies, Sirjan School of Medical Sciences, Sirjan, Iran; 2. Department of Environmental Health Engineering, Sirjan School of Medical Sciences, Sirjan, Iran

**Keywords:** COVID-19, culture, Iran, religion

To the Editor,

The outbreak of the new Coronavirus, known as COVID-19, in China in late 2019 and the subsequent rapid spread of the virus in various parts of the world has caused great concern in various countries. The high rate of virus spread has caused countries to face large numbers of infected people. As of April 13, 2020, 1.850 million people world-wide are infected with the virus. However, to date, more than 428,000 people have recovered. Yet, the incidence is still increasing. This infection occurs in less than three months. Also, the lack of definitive treatment has led to more than 119,000 deaths during this period.^1^

The outbreak of the virus in Iran was officially announced on February 20, 2020 in Qom Province. The virus quickly spread to all 31 provinces of the country. And as of April 13, 2020, it has infected nearly 72,000 people and killed 4,474. Currently, the incidence of morbidity and mortality in Iran is increasing, but the limitations and deprivations of international sanctions, the unknown nature of the disease, the inefficient use of information technology capacity in public opinion management, and the lack of an overview of the health issues cause the emergence of fear and emotional reactions in society.^2,3^

The quarantine approach in Iran was mostly based on interaction, and the Iranian government did not quarantine the city of Qom, despite public pressure, but only banned any rallies. The most important and wide-spread mass gatherings in Iran are religious mass gatherings around sacred religious sites. Despite pressure from religious groups, the government banned travel around these restricted areas to slow the spread of the disease. However, a group of religious people ignored the resolutions of the National Headquarters for Combating COVID-19 and spontaneously attended these shrines, and even licked the shrine, which is a very polluted place.^4^

Such behaviors in Iranian society highlight the need to pay attention to cultural challenges in controlling the epidemic of infectious diseases. Policymakers at the National COVID-19 Control Headquarters should use a community-based approach to epidemic control. The use of religious leaders to articulate approved policies is one of the most effective strategies in such situations. Such behaviors are a sign of a lack of education and, as a result, a low risk. The capacity of religious organizations themselves must be used to increase people’s understanding of the dangers of religious gatherings.^5^

Another issue that has been demonstrated in the adoption of COVID-19 control policies is the lack of risk governance in the country. The principles of sovereignty in Iran have not been well-implemented. In such a way, the military forces are used to implement the policies approved by the COVID-19 National Control Headquarters. Implementing various aspects of risk management in line with the priorities of the document is a necessity of risk management in Iran.^6,7^

In order to control the epidemic of infectious diseases in developing and less-developed countries, it is necessary to pay attention to cultural differences. Different strategies for engaging religious groups need to be studied, and programs are only effective if they take a community-centered approach.
